# The study on mat with hardness and elasticity for minimizing fatigue at various qork conditions

**DOI:** 10.1186/1757-1146-7-S1-A107

**Published:** 2014-04-08

**Authors:** Bo Seong Kim, Su Min Yoon, Hoon Yong Yoon

**Affiliations:** 1 Department of Industrial and Management Systems Engineering, Dong-A University, Busan, Korea

## Objective

This study was to investigate the changes by body part that occurred in a standing posture for a long time through EMG and Circumference of Leg. In addition, this study was to find out Mat that could minimize the degree of fatigue by body part according to the Elasticity and Hardness of Anti-Fatigue Mat by workplace circumstances.

## Background

There are many cases of working in the stands for a long time by form or by industry on the manufacturing sites and service industries, and in many cases, working in the stands happened in the spot of education and at home. There arose many body changes and a lot of loads to the lower limb and lumbar in a standing posture for a long time.

## Method

In order to identify the changes in the body the experiment was conducted by maintaining the standing posture for two hours by using three sorts of shoes state and four sorts of mats as an object of 9 test subjects. The Mat used in the experiment was designed on the basis of Elasticity 15% and 35%, Hardness 25Hs and 45Hs. As to the state of shoes, the experiment was progressed by dividing into three sorts of working environment: bare foot, wearing safety shoes and wearing slippers. The measuring of EMG was executed as an object of Erector Spinae, Rectus Femoris, Vastus Lateralis, Tibialis Anterior and Gastrocnemius Medialis. And, the Circumference of Leg, the circumference of crus was measured based on each projecting point of calf, and the circumference of thigh based on the center point of thigh.

## Results

The results showed that the elasticity, hardness, and interaction between elasticity and hardness have significant differences at the Erector Spinae, Rectus Femoris with barefoot (p<0.05). The elasticity, hardness, and interaction between elasticity and hardness have significant differences at the Rectus Femoris, Vastus Lateralis with safety shoes (p<0.05). The elasticity, hardness, and interaction between elasticity and hardness have significant differences at the Rectus Femoris with slippers (p<0.05).

